# Simultaneous and sequential hemorrhage of multiple cerebral cavernous malformations: a case report

**DOI:** 10.1186/s13256-016-0817-7

**Published:** 2016-02-09

**Authors:** Nundia Louis, Robert Marsh

**Affiliations:** Department of Neurosurgery, Cabell Huntington Hospital, Marshall University, 1600 Medical Center Drive, Huntington, WV 25701, USA

**Keywords:** Angioma, Cavernoma, Cavernous malformation, Hemorrhage, Hypertension

## Abstract

**Background:**

The etiology of cerebral cavernous malformation hemorrhage is not well understood. Causative physiologic parameters preceding hemorrhagic cavernous malformation events are often not reported. We present a case of an individual with sequential simultaneous hemorrhages in multiple cerebral cavernous malformations with a new onset diagnosis of hypertension.

**Case presentation:**

A 42-year-old white man was admitted to our facility with worsening headache, left facial and tongue numbness, dizziness, diplopia, and elevated blood pressure. His past medical history was significant for new onset diagnosis of hypertension and chronic seasonal allergies. Serial imaging over the ensuing 8 days revealed sequential hemorrhagic lesions. He underwent suboccipital craniotomy for resection of the lesions located in the fourth ventricle and right cerebellum. One month after surgery, he had near complete resolution of his symptoms with mild residual vertigo but symptomatic chronic hypertension.

**Conclusions:**

Many studies have focused on genetic and inflammatory mechanisms contributing to cerebral cavernous malformation rupture, but few have reported on the potential of hemodynamic changes contributing to cerebral cavernous malformation rupture. Systemic blood pressure changes clearly have an effect on angioma pressures. When considering the histopathological features of cerebral cavernous malformation architecture, changes in arterial pressure could cause meaningful alterations in hemorrhage propensity and patterns.

## Background

Cerebral cavernous malformations (CCMs), also known as cavernomas or cavernous angiomas, are classically defined as low pressure hamartomatous berrylike vascular lesions with minimal to no intervening brain parenchyma composed of thin-walled endothelial-lined sinusoidal spaces devoid of smooth muscle [1–4]. It has been suggested that CCMs arise from failure of vascular stabilization in angiogenesis which promotes the development of capillary dysplasia, weak intercellular junctions, and defective smooth muscle recruitment [5]. An estimated 0.5 % of the population has CCMs [3, 6]. While many patients remain asymptomatic, others tend to develop epilepsy, neurological deficits, or hemorrhage [1, 4, 6]. Sporadic and inherited forms of CCM have been described. The sporadic form often results in single isolated lesions while the inherited form is associated with multiple lesions and mutations of the endothelial genes *CCM1*, *CCM2* or *CCM3* [5, 7].

The etiology of CCM rupture is not well understood. It has been demonstrated that CCM lesions elicit inflammatory responses that involve tumor necrosis factor alpha (TNF-α) and interleukins (ILs). Upregulation of angiogenic factors such as vascular endothelial growth factor (VEGF) have also been described. These processes are implicated in the promotion of angiogenesis and breakdown of the blood–brain barrier (BBB) leading to the progression and rupture of CCM [5]. *CCM2* and *CCM3* mutations have also been linked to higher hemorrhage rates [1, 8]. Simultaneous hemorrhages of multiple CCM lesions are anecdotally common but few have been reported [7, 9, 10]. Causative physiologic parameters preceding hemorrhagic CCM events are often not described even in case reports. We present a case of an individual with simultaneous and sequential hemorrhages in multiple CCMs with a new onset diagnosis of hypertension.

## Case presentation

### History and examination

**Fig. 1:**
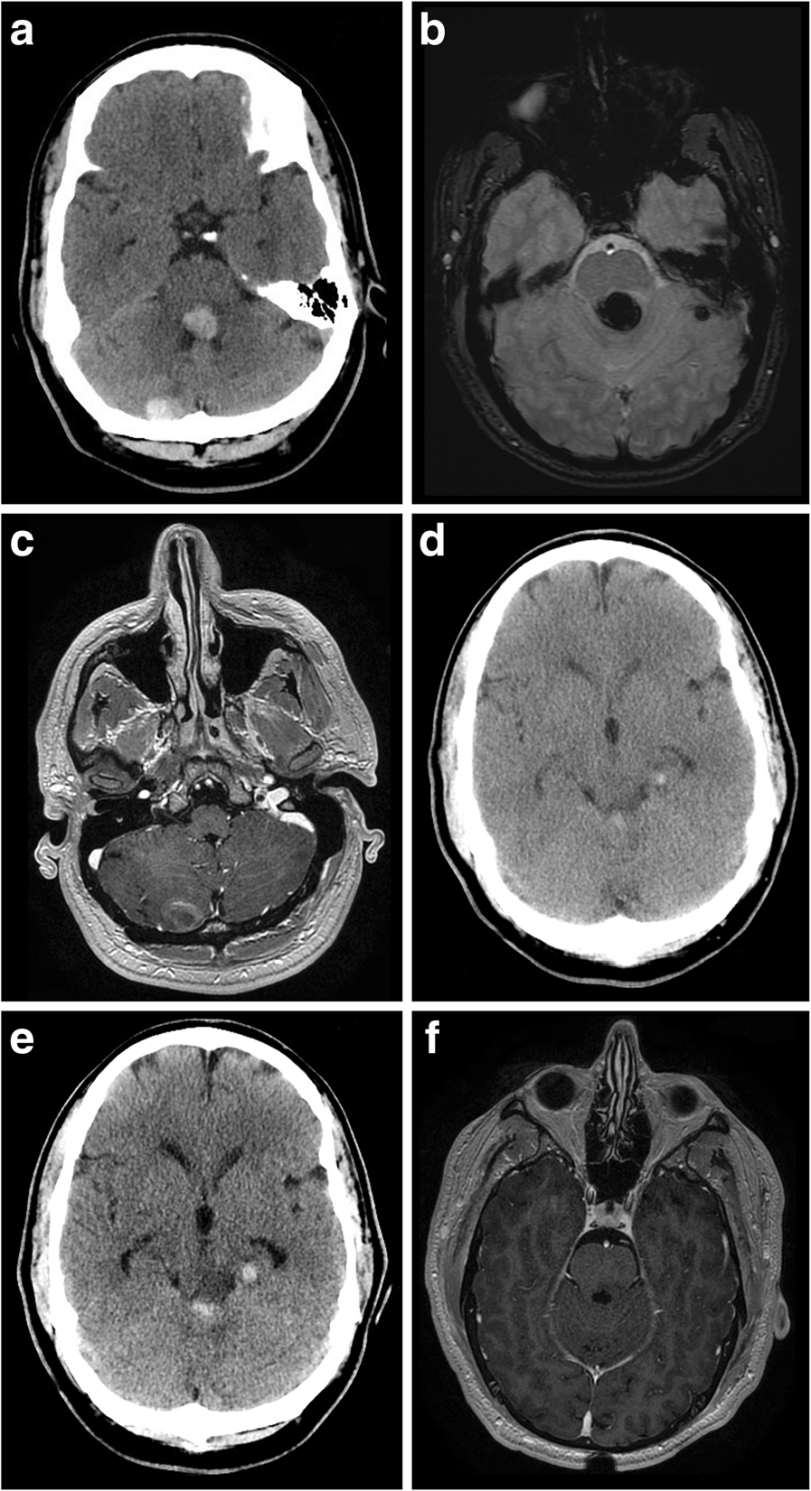
Cranial imaging of multiple cerebral cavernous malformations. **a** Computed tomography scan indicating two areas suspicious for acute hemorrhage: one within the fourth ventricle and the other adjacent to the calvarium in the patient’s right cerebellum. **b** Gradient echo magnetic resonance imaging showing lesion in left lateral cerebellar hemisphere. **c** Magnetic resonance imaging with contrast showing heterogeneous enhancement in the right cerebellar mass. **d** Computed tomography scan demonstrating increased hemorrhage and size within the ventricular lesion and the new hemorrhagic hyperdensity within the left medial temporal location. **e** Computed tomography scan displaying further hemorrhagic enlargement of the intraventricular and temporal lesions with development of hydrocephalus. **f** Postoperative magnetic resonance imaging showing complete resection of fourth ventricular and right cerebellar masses

We describe the case of a 42-year-old white man who was transferred to our facility due to worsening headache of 6 months evolution and new onset left facial and tongue numbness with dizziness. A head computed tomography indicated two areas suspicious for acute hemorrhage: one within the fourth ventricle and the other adjacent to the calvarium in his right cerebellum (Fig. 1a). His past medical history was significant for acute sinusitis and recent onset of hypertension. A physical examination at the time of presentation revealed blood pressure of 161/98 mmHg, ataxia, dysmetria, vertigo, and a positive Romberg’s test. Magnetic resonance imaging (MRI) was obtained with and without contrast.

The MRI showed two additional lesions: one in his left lateral cerebellar hemisphere and the other in his medial posterior left temporal lobe (Fig. 1b). All lesions exhibited significant signal dropout on gradient echo sequences. Heterogeneous enhancement was noted in the right cerebellar mass (Fig. 1c). The differential diagnosis included multiple cavernous malformations or hemorrhagic metastatic lesions.

Erythrocyte sedimentation rate, C-reactive protein, carbohydrate antigen 19-9, carcinoembryonic antigen, and a chest X-ray were ordered and found to be within normal limits. A computed tomography (CT) scan performed the next day showed increased hemorrhage size within the ventricular lesion and a new hemorrhagic hyperdensity within the left medial temporal location (Fig. 1d). The patient symptomatically improved over his 2-day hospital course with complete resolution of his dizziness and ataxia. He was discharged with orders for a repeat MRI after hemorrhage resolution and further testing. Surgical resection was delayed due to his presenting symptoms, uncertainty of etiology, and specific reports suggesting resection of CCMs after two bleeding incidents in eloquent brain regions or single hemorrhage in non-eloquent area accompanied by deteriorating neurological deficit [11].

The patient was readmitted 1 week later with headache, nausea, worsening dizziness, new onset diplopia and elevated blood pressure. He was found to have a fourth cranial nerve palsy, mild decrease in the right nasolabial fold, hypophonia and continued left facial and tongue numbness. A CT scan displayed further hemorrhagic enlargement of the intraventricular and temporal lesions with development of hydrocephalus (Fig. 1e).

### Operation

The patient underwent a suboccipital craniotomy and the right cerebellar lesion was resected first. The lesion demonstrated hemosiderin deposition with a gliotic margin. The telovelar approach was then used to access the fourth ventricle. The ventricular mass was well-circumscribed, pearly red and easily delineated from the ventral wall of the fourth ventricle. The mass was centrally debulked and the walls circumferentially collapsed. The gliotic margin adjacent to the brainstem was carefully delineated and gross total resection was achieved.

### Postoperative course

Immediately after surgery, the patient developed new onset mild left third nerve palsy. MRI showed complete resection of fourth ventricular and right cerebellar masses (Fig. 1f). Pathology confirmed diagnosis of CCM. He was discharged on postoperative day six with improved cranial nerve functioning and resolution of ataxia but continued vertigo. Approximately 1 month after surgery, his course was complicated by culture-negative bacterial meningitis and development of pseudomeningocele that resolved with aspiration and proper antibiotics treatment. He demonstrated complete resolution of ocular cranial nerve dysfunction but exhibited mild horizontal nystagmus with rotational challenge that resolved by 6 months.

## Discussion

Cerebral cavernomas are estimated to occur in 1 out of every 200 individuals in the general population [3, 6, 7]. They are hypothesized to develop due to failure of vascular stabilization in angiogenesis of cerebral blood vessels [5]. In a large series studying the natural history of cavernomas, 18.7 to 20 % of patients had multiple lesions [9, 12, 13]. Multiple lesions are mostly seen in familial CCM (FCCM) forms [7]. FCCM has been associated with mutations of *CCM1*, *CCM2* and *CCM3* genes. Nevertheless, 22 % of multiple lesions occur without any evidence of gene mutation [8].

CCMs have a hemorrhage rate of 0.7 to 1.1 % per lesion per year [4, 7]. Most cases of hemorrhages reported in the literature tend to involve one lesion, even in patients with multiple CCMs. Personal communication from Issam Awad suggests that multiple simultaneously hemorrhagic CCM lesions are more common (July 2015). However, to date there have been a total of only three reports on simultaneous hemorrhages of multiple cavernomas (Table 1). Our report is different in that the presented case exhibited simultaneous and sequential rupture patterns in the setting of new onset hypertension. What would lead to such a phenomenon remains an enigma, as the exact cause of CCM hemorrhage has yet to be determined. Many studies have focused on the genetic and inflammatory mechanisms contributing to CCM rupture, but few have investigated the potential role chronic hypertension may play in this complex multifactorial disease process.

**Table 1:** Details of the three published cases of simultaneous multiple cavernoma hemorrhages (and the present case)

Authors, year and reference number	Age (years), sex	Total number of cavernomas	Location of hemorrhagic cavernomas	Symptoms at time of presentation	Form of cavernoma	Surgical treatment	Follow-up
Panciani *et al.*, 2012, [7]	46, F	3+	1-Right posterior superior frontal gyrus 2-Left anterior cingulate gyrus	HA, NV and LUE paresis	Non-familial	Resection of frontal gyrus and cingulate gyrus lesions	10 days postoperative: residual hyposthenia
Chanda and Nanda, 2002, [9]	52, F	2	1-Dorsal midbrain region 2-Left occipital region	Ataxia, diplopia, and dysarthria	Not reported	Resection of midbrain lesion	Not reported
El Asri *et al.*, 2014, [10]	46, F	2+	1-Left occipital lobe 2-Right cerebellar hemisphere	HA	Not reported	Resection of left occipital and right cerebellar lesions	Not reported
Current case	42, M	4	1-Fourth ventricle 2-Right cerebellum 3-Medial posterior left temporal lobe	HTN, HA, dizziness, ataxia, left facial and tongue numbness, and diplopia	Not reported	Resection of ventricular and right cerebellar lesions	6 months postoperative: no residual deficit

*F* female, *HA* headache, *HTN* hypertension, *LUE* left upper extremity, *M* male, *NV* nausea and vomiting

Genetic and inflammatory causes have clearly been shown to influence CCM hemorrhage. In mouse model experiments, Cunningham *et al*. observed that conditional inactivation of the *CCM2* gene in adult mice produced a cerebral hemorrhage similar to that observed in adult human CCMs [1]. Mutation of the *CCM3* gene in humans has been linked to a hereditary variant of CCM and demonstrates early-onset cerebral hemorrhage patterns [8]. Shi *et al*. have reported CCMs to be active inflammation sites infiltrated with B cells and plasma cells [14]. Our patient did not have any familial past medical history of intracerebral hemorrhage or relatives with a diagnosis of CCM. However, he was being treated for sinusitis and it is possible that there was an increased release of inflammatory cytokines, TNF-α and ILs. These inflammation mediators stimulate angiogenesis and BBB breakdown and are thought to contribute to CCM rupture [5]. It is also possible that rupture of one of the CCMs enhanced recruitment of the inflammatory processes that contributed to the sequential pattern that was observed in our patient. However, inflammation and genetics may be only part of this complex multifactorial disease process and hypertension may play a role.

The studies that have addressed hemodynamic effects on CCMs are narrowly focused and limited. A recent study on the association of cardiovascular risk factors with CCM severity in 185 Hispanic individuals with *CCM1* mutation failed to find any positive correlation between CCM rupture and cardiovascular risks, including hypertension [15]. However, an experiment by Little *et al*. demonstrated that cavernomas are affected by changes in mean arterial blood pressure (MABP) and venous pressure. Direct angioma pressure measurements showed that a mean reduction of 14.7±2.1 mmHg in MABP resulted in a 7.0±0.5 mmHg drop in angioma pressure. Mechanical jugular compression induced real measurable changes in CCM pressure up to 9 mmHg [2]. Although the study did not investigate changes caused by increased MABP, the data clearly demonstrate that systemic blood pressure changes significantly affect CCM pressures.

Few studies to date have directly measured cerebral capillary pressure or determined the direct effects of systemic blood pressure on capillary physiology. Classical studies of cerebral blood flow show that the pial arterioles autoregulate until systolic blood pressure exceeds approximately 160 mmHg above which smaller pial arterioles are differentially affected, become dilated, and lose regulatory control; this results in increases in blood flow [16, 17]. Direct measurement of cerebral capillary pressure is problematic but pressure characteristics can be extrapolated from peripheral limb vasculature experiments which demonstrate that significantly higher apex pressures are measured in patients with essential hypertension when compared to age-matched and sex-matched normotensive controls [18, 19]. Structural abnormalities can also occur in peripheral capillaries as a result of essential hypertension with loss or reduction in the density of vessels per volume of tissue, which is a process known as rarefaction [18, 20]. When considering the histopathological features of CCM architecture, chronic hypertensive changes alter arterial flow and vessel physiology, which could cause meaningful alterations in capillary anatomy as well as hemorrhage propensity and patterns.

Many vascular disease processes are influenced by multifactorial systemic corporeal changes. An example of this is the development and rupture of cerebral aneurysms. Inflammation, genetics, hypertension, smoking, and age are known risk factors that contribute to the development of aneurysm rupture and hemorrhage [21–25]. Models have been developed to describe the pathophysiology for aneurysm induction and progression and include endothelial damage and degeneration of the elastic lamina, inflammatory cell recruitment and infiltration, and chronic remodeling of vascular wall [26]. Similarly, we argue that chronic high blood pressure may be a factor in capillary physiology that alters architectural features of abnormal capillary anatomy in CCM which increases the propensity for hemorrhage.

## Conclusions

This case report is unusual in that hypertension may have played a role in the simultaneous and sequential hemorrhage pattern noted in this individual. Notwithstanding the many advances made in understanding the structure, formation and evolution of CCMs, there is still a lack of understanding concerning the effect of hemodynamic changes on cerebral capillary physiology and cavernomas. Investigative work focusing on the role of hypertension and other hemodynamic factors in CCM rupture is much needed, especially experiments in order to better determine if blood pressure changes affect the incidence of CCM hemorrhage.

## Consent

Written informed consent was obtained from the patient for publication of this case report and accompanying images. A copy of the written consent is available for review by the Editor-in-Chief of this journal.
